# Modeling the Visual Landscape: A Review on Approaches, Methods and Techniques

**DOI:** 10.3390/s23198135

**Published:** 2023-09-28

**Authors:** Loukas-Moysis Misthos, Vassilios Krassanakis, Nikolaos Merlemis, Anastasios L. Kesidis

**Affiliations:** 1 Department of Surveying and Geoinformatics Engineering, University of West Attica, GR-12243 Athens, Greece; lmmisthos@uniwa.gr (L.-M.M.); krasvas@uniwa.gr (V.K.); merlemis@uniwa.gr (N.M.); 2 Department of Public and One Health, University of Thessaly, GR-43100 Karditsa, Greece

**Keywords:** landscape perception, landscape evaluation, landscape characterization, egocentric/exocentric landscape perspectives, sensor-based experimental techniques, eye tracking, fMRI, EEG, GIS, landscape quantification and modeling

## Abstract

Modeling the perception and evaluation of landscapes from the human perspective is a desirable goal for several scientific domains and applications. Human vision is the dominant sense, and human eyes are the sensors for apperceiving the environmental stimuli of our surroundings. Therefore, exploring the experimental recording and measurement of the visual landscape can reveal crucial aspects about human visual perception responses while viewing the natural or man-made landscapes. Landscape evaluation (or assessment) is another dimension that refers mainly to preferences of the visual landscape, involving human cognition as well, in ways that are often unpredictable. Yet, landscape can be approached by both egocentric (i.e., human view) and exocentric (i.e., bird’s eye view) perspectives. The overarching approach of this review article lies in systematically presenting the different ways for modeling and quantifying the two ‘modalities’ of human perception and evaluation, under the two geometric perspectives, suggesting integrative approaches on these two ‘diverging’ dualities. To this end, several pertinent traditions/approaches, sensor-based experimental methods and techniques (e.g., eye tracking, fMRI, and EEG), and metrics are adduced and described. Essentially, this review article acts as a ‘guide-map’ for the delineation of the different activities related to landscape experience and/or management and to the valid or potentially suitable types of stimuli, sensors techniques, and metrics for each activity. Throughout our work, two main research directions are identified: (1) one that attempts to transfer the visual landscape experience/management from the one perspective to the other (and vice versa); (2) another one that aims to anticipate the visual perception of different landscapes and establish connections between perceptual processes and landscape preferences. As it appears, the research in the field is rapidly growing. In our opinion, it can be greatly advanced and enriched using integrative, interdisciplinary approaches in order to better understand the concepts and the mechanisms by which the visual landscape, as a complex set of stimuli, influences visual perception, potentially leading to more elaborate outcomes such as the anticipation of landscape preferences. As an effect, such approaches can support a rigorous, evidence-based, and socially just framework towards landscape management, protection, and decision making, based on a wide spectrum of well-suited and advanced sensor-based technologies.

## 1. Introduction

### 1.1. General Background

Humans have evolved in close interaction with their surrounding natural environment [1]. Our survival and well-being depend on actively experiencing and engaging with the landscape around us [2,3,4,5]. As vision has become the dominant human sense [6], the composition and spatial configuration of landscape elements, relative to our field of vision have become crucial for our survival and thriving [7,8,9,10].

Landscape perception depends heavily on human vision [11,12,13]. The ‘ocular toolkit’ of vision and, particularly, the eyes can be considered as a compound biological light-sensitive sensor and system [6]. This system enables the separation of spectral—that is luminance and chromatic (color)—information in the visual system [14], while it also facilitates the encoding and representation in the human eye retina of both high-resolution spatial (‘achromatic’) and lower resolution color (‘chromatic’) signals [15]. Ungerleider and Mishkin [16] have suggested two separate pathways or streams (and cortical areas) by which the visual information is processed: the ventral stream which mediates the recognition of objects (‘what’ an object is) and the dorsal stream which mediates the objects’ spatial configuration identification (‘where’ an object is) [16,17]. Later on, Goodale and Milner [18] put forward a modified version of the “what/where” model, proposing a similar distinction between “perception” versus “action”, for ventral and dorsal processing streams, respectively [17,18]. Recently, the clear-cut separation of the “what/where” model has been challenged, mainly because the ventral stream’s role is not only confined to object recognition, and because the dorsal stream is not solely responsible for spatial vision; it also contributes to visual and spatial attention [19]. As an affect, the dorsal stream is mainly engaged in the visual “exploration” of our environment, while the ventral stream is engaged in the “exploitation” of a focused area/part of our environment [19].

**Figure 1:**
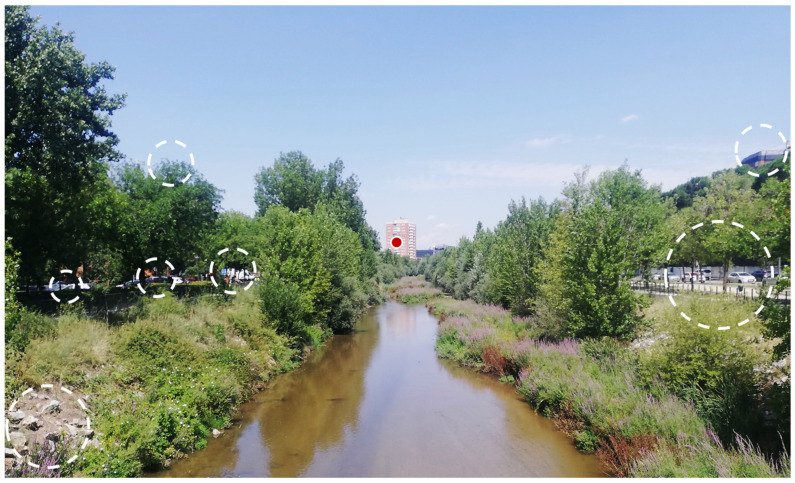
Illustration of the separate functions of the dorsal and ventral pathways/streams during the observation of a landscape scene: the dorsal stream mainly engages with the entirety of salient areas all over the visual field (hollow circles with white, dashed outlines), and the ventral stream emphasizes the central part of the visual field (red circle with white outline in the center of the figure). The figure has been created according to [19].

In a nutshell, in landscape visual experience, both streams process our surroundings, executing different but complementary functions or tasks. While the dorsal stream sets under process all of our visual space, the ventral one emphasizes the central/focal part of this field; “salient, moving and/or changing parts of the scene in the visual periphery are vying for the observer’s focus and attention, and decisions are being made on where in space to move the eye.” [19] (p. 37). Figure 1 illustrates how the two functions/tasks interact while viewing a landscape image or scene. Functions related to the dorsal stream are suited to coarsely explore the entirety of the landscape, detecting stimuli and features occurring all over the landscape, while at the same time executing plans to bring stimuli and features into focus for further, more refined processing through functions related to the ventral stream; such functions are particularly appropriate for extracting structured and high-level information about stimuli and landscape features located at the center of focus [19].

The exact behavioral tasks and their explicit interconnections with the responding visual pathways/streams and cortical areas are not thoroughly examined in this review article. Yet, the distinctions made in the aforementioned paragraphs are a prerequisite in order to further proceed to the modeling of landscape visual experience (perception) from the *human perspective*, which is considered by several researchers an essential endeavor (e.g., [20,21,22,23]). Towards this direction, several research studies have recently been dedicated to the experimental recording and quantification of landscape *visual perception*, using mainly methods and sensor-based techniques known as *eye tracking* and *eye movement analysis* (e.g., [24,25,26,27,28,29,30,31,32,33,34,35,36,37,38,39,40,41,42]). Other research studies have employed other sensor-based experimental techniques and technologies to register and quantify the brain activity during the observation of landscapes (scenes) such as *functional Magnetic Resonance Imaging* (*fMRI*) (e.g., [43,44,45,46,47]) and *Electroencephalography* (*EEG*) (e.g., [48,49,50,51,52,53]).

A multitude of other scientific studies have focused—in the last four decades or so—on how to register and quantify visual landscape *evaluation* (*or assessment*), mainly using participants/correspondents who observe and rate landscape photographs, by employing several empirical techniques (e.g., [54,55,56,57,58,59,60,61,62,63,64,65,66,67,68,69,70,71,72,73,74,75,76,77]). A recent review article systematically describes and classifies existing landscape evaluation approaches and methodologies adopted, along with indicators developed/selected and technologies used [78].

**Figure 2:**
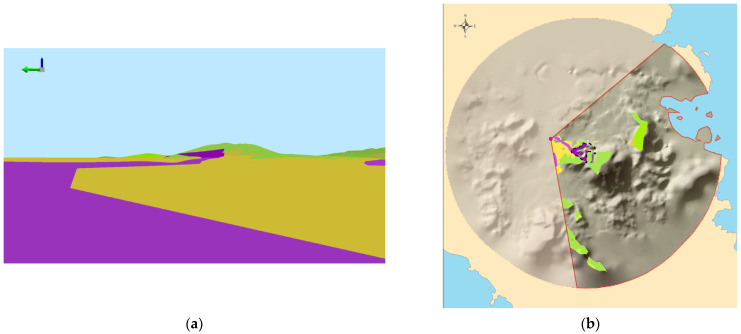
Graphical illustration of the two meaningful perspectives/visualizations under which a landscape can be conceived: (**a**) Egocentric perspective; (**b**) Exocentric perspective. Adopted from [21].

The visual landscape can be approached/modeled either from the human (ground) standpoint, i.e., the way people experience their surroundings in everyday life (as they stand, walk, etc.), or from a standpoint whereby land is vertically projected from above, i.e., the way maps portray parts of the landscape (in cartographic products and geovisualizations). The first perspective is defined as *egocentric* while the second one as *exocentric* (e.g., [21,23,79,80,81]). Figure 2 provides an illustration depicting how the same (abstracted) visual landscape can be conceived and rendered under two meaningful, alternative perspectives/visualizations; Figure 2a portrays the egocentric perspective, whereas Figure 2b portrays the exocentric perspective (for more details, see [21,23]).

Perhaps the most well-established scientific ‘discipline’ or tradition that adopts the exocentric approach in landscape research is that of Landscape Ecology. Within this tradition, landscapes are considered extensive land mosaics, “over which local ecosystems recur” [82] (p. 20). The inherent structure and the spatial patterns of the ecosystems within a landscape are intertwined with the function and ecological processes of these ecosystems [7,83,84,85,86], while landscape description and analysis are based on metrics and indices quantifying the planimetric (and vertical) heterogeneity of landscape’s constituent elements [7,8,83,87,88]. In this sense, landscapes are considered as mind-independently defined geographical entities (i.e., “ecological meaningful units” [89]) characterized by intrinsic heterogeneity (diversity of landscape elements), being described by an exocentric perspective. As a consequence, according to this tradition, landscapes can be objectively analyzed, without the implication of human presence/perception, while criteria, methods, and techniques have been proposed and developed in order to analyze/classify/assess the character of landscapes mainly in Europe, or elsewhere [89,90,91,92,93,94,95,96].

As mentioned above, these two perspectives are connected to different scientific traditions, as well as to different methods, techniques, indices and metrics, (sensor-based) technologies, and software tools for registering, recording, quantifying, and visualizing the (possible experience of the) landscape. Thus, visual landscape perception and evaluation are, in fact, and can be potentially modeled in different ways, depending on the perspective; even more fundamentally, they have different meanings.

### 1.2. Identification and Outline of the Research

This review article is dedicated to describing and distinguishing the different ways the landscape is modeled; it mainly aims to showcase and classify—among a staggering battery of relevant research studies—the dominant different/alternative approaches for modeling the visual landscape, promoting integrative frameworks for these approaches. Through an extensive literature review, primarily based on scientific article search engines (e.g., Google Scholar, Scopus, etc.) and with an emphasis on the last 10 years, existing approaches to visual landscape quantification and modeling are identified in a two-fold manner, namely based on the following: (1) Whether the focus is on landscape perception or landscape assessment/evaluation; (2) Whether the landscape is observed from an exocentric or egocentric perspective. According to our viewpoint and other researchers in the field of landscape research, even though these two dualities seem divergent for several reasons they are somehow complementary (e.g., [20,23,97,98,99,100,101]). Therefore, in this article, the theoretical and practical aspects of these two dualities—namely the perception/evaluation modalities, and the exocentric/egocentric perspectives—are embedded in an integrative framework.

Aside from presenting the main constituent parts of this dual approach, the article also delineates and provides a cohesive structure for the different theoretical, methodological, and technical/technological approaches for representing, registering, recording, and quantifying/modeling the (possible experience of the) landscape. Since the two different perspectives and the two different modalities (i.e., perception or assessment/evaluation) are by definition linked to different concepts and registrable/quantifiable elements, the (experimental or not) techniques, technologies, and software tools also differ.

Towards this end, the extensive examination of established approaches to landscape perception/description and evaluation, from the two perspectives, with a particular focus on supporting methods, techniques and technologies takes place in Section 2 and Section 3: Section 2 provides a theoretical analysis of the distinction between perceptual and evaluative aspects (i.e., the two modalities) regarding the visual landscapes, while it also provides a description of scientific approaches dedicated to the perception and evaluation of the visual landscape via the egocentric perspective; in Section 3, after analyzing the differences in conceiving the landscape via the two ‘divergent’ perspectives, we critically present how landscape is descriptively and normatively approached within the framework of the exocentric perspective. In Section 4, the findings from our literature review are summarized and discussed; the majority of the landscape research studies examined in this article are aggregated and classified in a meta-analysis guiding table: the examined ‘activities’ related to landscape experience under the egocentric/exocentric perspectives are linked to specific meanings, keywords, stimuli, sensors, metrics, and literature; additionally, crucial topics regarding the challenges and perspectives of integrating the two dualities (perspectives and human modalities) are critically discussed. Concluding remarks and potential future prospects are also presented in Section 4.

## 2. The Two Modalities of Approaching the Visual Landscape: Concepts, Methods and Metrics

Exploring whether perception involves concepts and/or cognition (i.e., knowledge) has been a subject of acute philosophical debate in the tradition of the philosophy of mind [102,103,104,105,106,107,108,109,110,111] and a subject of scientific debate in the tradition of cognitive science [112,113,114,115,116,117]. *Early vision* (*system*) is a very crucial term in this debate, regarding the cognitive (im)penetrability of perception. Early vision can be defined as being “identical to the pure bottom-up (stimulus-driven) visual processes”—corresponding “to the first neural processing stages in the retina and the visual cortex”—“without being influenced by the top-down stream of semantic information” or by the “higher cognitive and semantic cues” [118] (p. 740). According to [112] (p. 343), “if a system is cognitively penetrable then the function it computes is sensitive, in a semantically coherent way, to the organism’s goals and beliefs, that is, it can be altered in […] relation to what the person knows”. The defenders of cognitive impenetrability of perception argue that during early vision—that is, for the first milliseconds in which only bottom-up processes take place—visual perception is not influenced by cognition (e.g., [112,116]); as a result, the way one perceives a visual scene, or the world as whole, is distinct from her/his beliefs, expectations, evaluative judgements, etc. This postulation has been refuted by several scholars and researchers, resorting mainly to the fact that visual perception is not limited to early vision [117], and that the actual phenomenology of visual experience cannot be described and explained only by the content (i.e., representations) of early vision [115]. This divergence between researchers who adopt a *bottom-up* approach, focusing on the rich array of stimulus from the external world, and those who advocate a *top-down* approach, emphasizing the cognitive functions inside the brain (e.g., perceiver’s expectations and previous information) in constructing a likely account of what is out there has been characterized by [119] (p. 25) as the “major theoretical divide”.

In the realm of landscape research, perception and cognition are also present as distinct concepts. The plethora of definitions and meanings for landscape reveals the ambiguity for delineating what a landscape is. Landscapes carry multiple meanings (e.g., [120]) which can, for example, refer to a bordered territory, a territorial identity, a scenery, an expression of ideas, etc. [121,122]. More concisely, Unwin [123] (p. 130) defines landscape as “the appearance of the land at the interface of the earth’s surface and atmosphere”. In the noted definition of the European Landscape Convention (ELC), a landscape is: “an area, as perceived by people, whose character is the result of the action and interaction of natural and/or human factors” [124]. According to alternative approaches, landscape is conceived as a process or interaction rather than as an object or a finalized product [125,126], “subjectively ‘in the making’ rather than as an assemblage of physical features” [126] (p. 119). “Landscape experience, then, is not just how a given view comes to be represented, but how its viewer stakes a claim to perception and to presence” [127] (p. 104).

The same ambiguity arises when it comes to the definition of landscape perception. Dupont [122] identifies three major meanings for visual perception: (1) The first one refers just to visual sensation: that is the physiological process of collecting information from the environment through senses (e.g., sight (vision)); (2) The second one extends this physiological process, by attaching to sensation other mental or cognitive processes such as understanding or interpretation; (3) The third one solely refers to interpretation or understanding processes, pointing to mental states such as opinions or beliefs.

**Figure 3:**
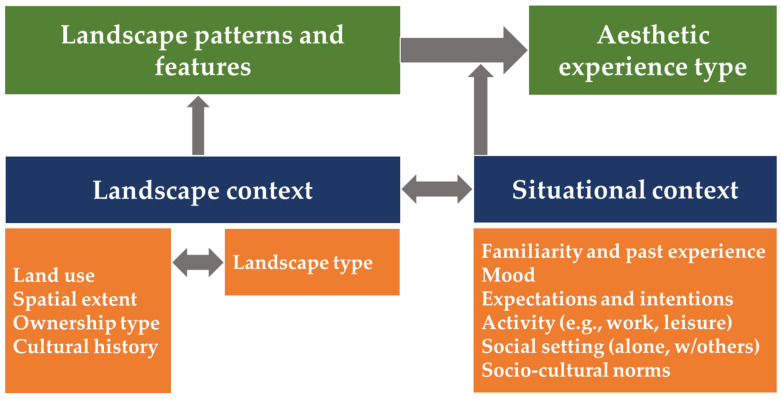
Abstracted model of interactions taking place between the landscape context and the situational context towards the emergence of landscape aesthetic experience. The figure has been created according to [128].

As far as our everyday landscape experience encompasses both a physiological and a psychological (cognitive) component (second meaning), landscape visual perception and landscape evaluation are modalities that may also appear indistinguishable. Landscape observation is influenced by *cognition*, while landscape evaluation requires the existence of a *perceivable* area of land; however, several methods and techniques for registering these two different modalities have been developed—albeit, it is not always clear which exact modality is registered each time. As Gobster et al. [128] have pointed out, the aesthetic experience type with regard to the landscape emerges from the interaction between the following: (i) The “landscape context”, referring to the different geometric patterns and features of the landscape—potentially eliciting attention to different visual patterns; (ii) The ‘‘situational context’’, referring to the socio-cultural and personal factors influencing the observer (Figure 3).

For reasons of functionality, landscape perception and evaluation of the visual landscape are delineated in this article as such:

- Landscape perception is by and large equivalent to the occurring behavioral patterns of visual activity and/or brain activity, while visually experiencing a landscape (scene);
- Landscape evaluation refers mainly to the people’s judgements or appraisal of landscape, meaning landscape (visual quality) preferences and ratings, while visually experiencing a landscape (scene).

In addition, the concept of the landscape itself is adumbrated in the context of this review article. To this end, the two alternative perspectives—namely the exocentric and the egocentric perspectives (see also Figure 2 and Section 3.1)—which generally coincide with the two main definitions of the Landscape Ecology and from the ELC (European Landscape Convention), are taken into consideration. Therefore, the landscape is conceived as both an area of land having *inherent* content (landscape elements) and spatial properties (relationships among elements) and a *view* or *vista* having the (human) observer as a point of reference. In this sense, the quantitative and qualitative content and properties of landscapes *per se* are considered, albeit putting emphasis on how landscape content and properties can be visually perceived (see [20,21,23] and Figure 2). As a consequence, the landscape scaling in this article ranges from wider geographic ranges, such as the regional- or even national-level (e.g., [86,89,91,92,95]), narrowed down to the scaling of the views than the human observation/vision can afford (e.g., [9,11,20,21,23]).

**Figure 4:**
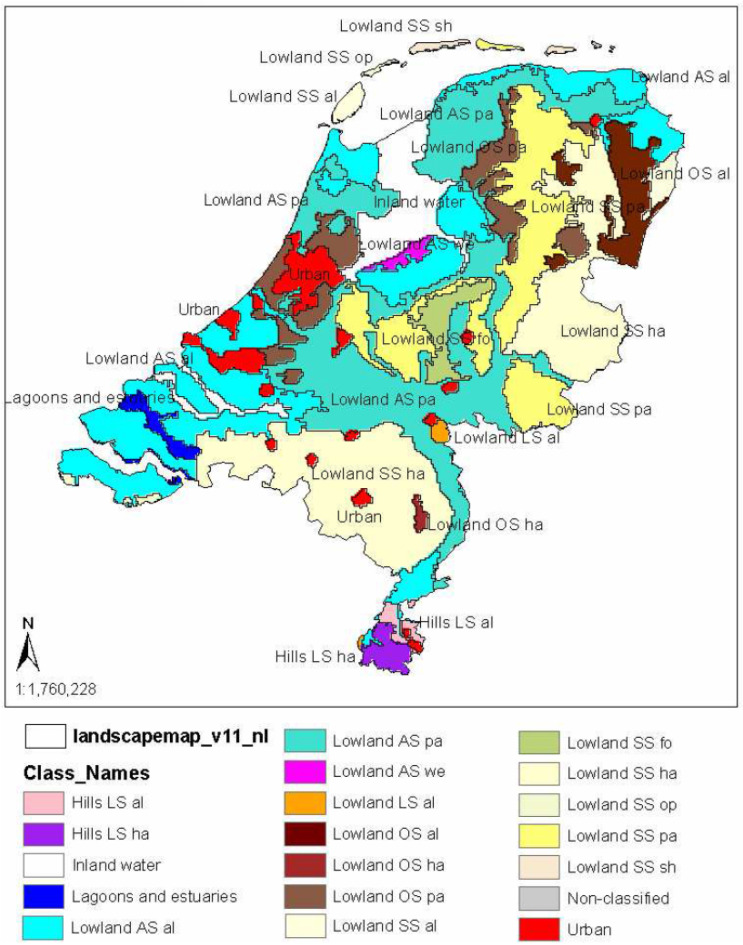
Examples of large landscape/geographic units included in the LANMAP classification methodology: the landscape types (e.g., Hills LS al, Lowland OS ha, etc.) that are cartographically represented occur due to the combination (overlaying) of suitable input geospatial data (e.g., altitude, land use, etc.). Figure adopted from [95].

**Figure 5:**
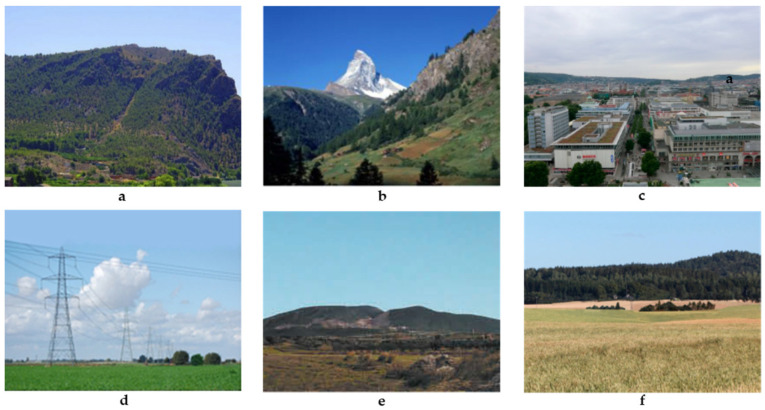
Examples of landscape types represented as visual scenes (photographs or photographic simulations) occurring in everyday life: (**a**) Forest [55]; (**b**) Mountainous [44]; (**c**) Urban [26]; (**d**) Industrial [129], (**e**) Mining [29]; (**f**) Rural/agricultural [130].

The range of ‘eligible’ landscapes or landscape types considered in this article is also wide: from the large geographic units that are cartographically represented in maps created by landscape classification methodologies, such as LANMAP(2) [89,91] (Figure 4), up to the visual scenes occurring in the everyday life, characterized as ‘urban’, ‘rural’, ‘forest, ‘mountainous’, ‘industrial’, ‘mining’ landscapes, etc. (e.g., [26,29,44,55,129,130]) (Figure 4). Figure 4 and Figure 5 provide indicative landscape (type) examples at the two ‘ends of the spectrum’ of landscape conception, respectively.

### 2.1. Methods and Techniques for Modeling Visual Landscape Perception

#### 2.1.1. Eye Tracking and Eye Movement Analysis

Modeling visual perception, in the sense of recording and quantifying the patterns of *visual attention*, can be achieved by employing eye tracking methods and techniques. Eye tracking is one of the most popular experimental approaches used to examine both visual perception and cognition (e.g., [131,132,133,134,135]). Eye tracking techniques and methods are used to monitor and analyze eye movements during the observation of visual or audiovisual stimuli. More specifically, eye tracking techniques are implemented to measure the position of the eyes relative to the head of an observer and, more precisely, to capture the position of the eyes in space (the so-called “point of regard”). Over the last few years, several techniques have been developed to support these aims, including Electro-Oculography (EOG) (e.g., [136]), the utilization of search coils and contact lens (e.g., [137]), Photo-Oculography (POG) (e.g., [138]), and Video-Oculography (VOG) (e.g., [139]). Modern eye tracking devices, including low-cost solutions, mostly employ video-based methods that are based on pupil and corneal reflection and use cameras and infrared (IR) sensors [135]. Moreover, existing webcam-based, eye-tracking solutions now provide the opportunity to perform experimental studies remotely (e.g., [140]). Although the spatial accuracy of webcam eye tracking solutions is generally lower than that provided by state-of-the-art devices, such solutions can be considered adequate for many applications [141]. Furthermore, several research studies (e.g., [142]) indicate that webcam-based eye tracking could be suitable in the near future for performing scientific experimentation in cognitive sciences.

The main output of an eye tracking recording procedure is the spatiotemporal coordinates of gaze produced during the observation of a visual scene. A visual scene can be any visual stimulus presented on a monitor (including remote devices such as smartphones or tablets), the real space, or a virtual environment. Additionally, modern eye tracking devices can record binocular gaze data and measure fluctuations in pupil diameter (pupillometry [143]) during the observation of a visual stimulus. The collected eye tracking protocols are the spatiotemporal gaze data that are analyzed using both fundamental and derived metrics [144]. Fundamental eye tracking metrics refer to two different types of eye movements; fixations and saccades. During a fixation event, eyes remain relatively stationary to a specific point of the visual scene [145,146,147]. In practice, eye movement analyses consider fixation centers to represent the gaze data that correspond to the fixational movements. The transitions between fixation points (fixations) correspond to the saccadic movements (saccades), while the basic derived metric that is produced by the sequence fixation–saccade–fixation, etc., is called the scanpath [133,145,146,147].

Eye movement quantitative analyses include the development of specific metrics/indices based on fixations, saccades, and scanpaths (e.g., [148]). Such indices are adapted to the research needs of the examined domain. Specific or more sophisticated (i.e., compound) indices are developed in order to support research in specific scientific fields (see e.g., the indices developed by [29,149] in the framework of landscape research). Additionally, eye movement quantitative analyses are qualitatively supported by implementing several visualization techniques [150,151] that aim to depict either the individual visual search strategies (e.g., [152]) or the corresponding aggregated (cumulative) visual behavior (e.g., [153]). Several software tools, including both commercial and open source solutions (see e.g., [154,155]), have been provided by the scientific community to support the quantitative and the qualitative analyses of eye movements.

Recent review studies are dedicated to the application of eye tracking technologies in domains related to landscape research, such as cartography [156,157] and spatial research [158]. Yet, to the best of our knowledge, there is no such review study dedicated to landscape research itself, even though a quite large number of research papers exists, having employed eye movement recording and analysis techniques (e.g., [24,25,26,27,28,29,30,34,35,38,39,40,41,42,159,160,161]), as previously mentioned. For instance, Dupont et al. [42] have utilized the eye movement indices: number of fixations, number of saccades, and scanpath length; through this experimental study, it was mainly shown that for landscape photographs characterized by greater levels of urbanization (and by greater visual complexity), observers tend to exhibit more dispersed viewing patterns in order to assimilate the greater possible amount of visual information. In the research article of Misthos et al. [29], the effect of two variables was experimentally tested: position and apparent size of quarries in mining landscape photographs. Three eye movement indices were utilized: time to first fixation within the quarry, number of fixations within the quarry compared to the total number of fixations, and number of fixations within the quarry compared to the total number of fixations per each photograph. It was experimentally shown and statistically corroborated that observers’ viewing patterns are influenced by the two variables (position and size of the quarry). In the research study of Misthos and Menegaki [33], the eye movements of observers viewing different mining landscapes were visualized utilizing attention heatmaps; these heatmaps showed the clear visual dominance of quarries (excavated surfaces) in the landscape, while by comparing these visualizations with the results from computational models of attention allocation, i.e., *saliency models/maps* [40,162], it occurred that in some cases (types) of mining landscapes the theoretical prediction of visual attention allocation is effectible.

#### 2.1.2. fMRI and EEG

In addition to modeling behavioral patterns of visual activity, there are other quantitative methods for registering and quantifying brain/cortical activity while performing everyday tasks, such as observing a visual scene. Two widely used techniques for studying brain function are functional magnetic resonance imaging (fMRI) and electroencephalography (EEG). fMRI is a non-invasive technique that uses magnetic fields and radio waves to measure brain activity by detecting changes in blood flow and oxygenation, which are closely correlated with neuronal activity. As an effect, researchers using fMRI techniques are capable of mapping brain activity in real-time. EEG is also a non-invasive technique which measures the electrical activity of the brain using sensors placed on the scalp. The electrical activity is reflected in voltage fluctuations of the scalp electrodes, which can be recorded and analyzed to study brain activity.

One of the main advantages of fMRI is its high spatial resolution at the sub-millimeter level, enabling researchers to identify brain activity with high sensitivity and specificity [163,164,165]. However, fMRI has low temporal resolution, rendering difficult the study of rapid changes in brain activity and the capturing of fast cognitive subprocesses. In contrast, EEG has higher temporal resolution since it can detect rapid changes in brain activity in the order of milliseconds, although its spatial resolution is relatively low. EEG is relatively low-cost and easy to use, making it more accessible to researchers compared to other techniques such as fMRI, positron emission tomography (PET), and magnetoencephalography (MEG), which are more expensive and require specialized equipment. EEG and fMRI are often used in combination; for example, ultra-high field fMRI and high-density EEG have been recently utilized to study the ongoing thoughts of participants in the experiment and the spatiotemporal dynamics of their mentation [166]. fMRI techniques have also been proposed for use in combination with eye tracking [167]. fMRI, EEG and eye tracking have been recently utilized in the research of perceptual and cognitive influences of architectural and urban environments [168].

Image processing indicators are often needed to analyze fMRI data in order to extract meaningful information about brain activity and understand the functional organization of the brain, such as Independent Component Analysis (ICA), seed-based functional connectivity analysis, and graph theory. Clusterwise ICA has been proposed for discovering neurofunctional subtypes from multi-subject resting-state fMRI data [169]. In order to understand the spatiotemporal dynamics of brain activity, EEG data are analyzed using image processing indicators that include time-frequency analysis, event-related potentials (ERPs), and spatial filtering techniques. In recent years, machine learning techniques have also been applied to the analysis of fMRI and EEG data, with the aim of automating the detection and classification of brain activity patterns. Wen et al. present a brief overview of deep learning methods to process fMRI data [170], while deep learning techniques such as convolutional neural networks (CNNs) have been used to classify brain activity and to identify functional brain networks [171]. Other machine learning techniques, such as support vector machines (SVMs) and decision trees, have also been applied to fMRI data analysis with the aim of improving the accuracy and efficiency of brain activity classification. These techniques have the potential to significantly enhance the capabilities of fMRI as an image indicator, and to provide new insights into brain function in the case, for example, of the recognition of visualized objects from fMRI data [172]. Machine learning techniques have also been applied to the analysis of EEG data in recent years. Deep learning techniques such as CNNs and recurrent neural networks (RNNs) have been used to classify brain activity and to identify functional brain networks (e.g., [173]). SVMs and decision trees have also been applied to EEG data analysis, with the aim of improving the accuracy and efficiency of brain activity classification [174].

In the case of visual perception quantification, both techniques have met an increased interest in recent years, e.g., for the study of the spatio-temporal dynamics of face perception utilizing SVMs decoding and representational similarity analysis [175]. Information on brain visual selection based on combined data from fMRI, ERPs, and transcranial magnetic stimulation (TMS) is thoroughly discussed by Theeuwes [176]. More specifically, the brain activities of landscape perception and cognition have been recently explored by applying fMRI (e.g., [43,44,45,46,47]) and EEG techniques (e.g., [48,49,50,51,52,53]). It should be noticed that when utilizing these techniques it is more difficult to discern if the visual perception or cognition are modeled. In many of these studies, both perceptual and cognitive or emotional aspects are modeled.

For instance, Tang et al. [44] employed fMRI techniques during the observation of four different types (i.e., urban, mountain, forest, and water), and found out that, in terms of neural responses, different landscape types do have different effects on brain region activity; these fMRI-driven results were then compared to the results of a questionnaire-based psychological study, whereby a perceived restorative value was assigned to each of the four landscape types. However, in the fMRI research study of [43], the main aim was to identify the neural basis underlying both the perception and appreciation of natural landscapes and landscape gardens by properly manipulating the brain regions that were activated. fMRI has also been utilized to investigate the visual perception and aesthetic appeal of dynamic natural landscapes (i.e., movies) [47] and to provide evidence that visual processing and emotion-related regions of the brain are more active under dynamic landscapes viewing [46].

Regarding EEG, the research work of [49] aimed at assessing the accuracy of landscape perception and recognition of some typical landscape types (e.g., forest, wetland, and farmland) based on different EEG features and different classifiers. Furthermore, Wang et al. [53] investigated the role of color and structure in landscape recognition by using the objective quantitative index of EEG features and ended up choosing a specific classifier (SVM) to properly study landscape recognition and aesthetics [53]. Roe et al. [48] utilized objective indicators based on EEG data to detect emotional changes while viewing ‘green’ and ‘gray’ landscape scenes; these indicators were found to be consistent with subjective preferences, as well as with restorative theory. Visitor visual perception and attention of a forest landscape have been recently investigated utilizing a mobile EEG methodology, while the results of EEG attentional changes were coupled with questionnaire-based data regarding the perceived visual quality of forest landscapes, as rated by experts [52].

### 2.2. Methods and Techniques for Modeling Visual Landscape Evaluation/Assessment

As previously stated, landscape assessment/evaluation is a process that is distinct from landscape perception, albeit not entirely independent from it. Since the 1960s, landscape-related scientific research carried out by geographers, forest scientists, ecologists, psychologists, etc., has focused on developing standardized procedures for visual quality evaluation to establish and/or ensure the protection of environmentally significant and/or fragile areas (e.g., national parks and national reserves) [78]. In the same vein, early landscape evaluation was typically referred to as “visual assessment of landscape quality” [78], supported by influential visual/landscape resource management systems (VMS, VRM, and LMS) being proposed in USA [177,178]. In the 1980s, there was a more systematic attempt by researchers dealing with landscape (aesthetic) evaluation to “explicitly separate the classification and description of landscape attributes in order to distinguish the difference between the landscape of one area and another area” [78] (p. 109174). Later on, the focus of research was directed towards the effect of landscape features/characteristics on landscape rating and evaluation: such features were treated as “essential factors influencing the appraisal of landscape visual quality” [78] (p. 109174).

Landscape evaluation/assessment mainly focuses on registering psychological or behavioral preferences of different landscapes or landscape types, based on landscape features by employing empirical (qualitative or quantitative) methods. Daniel and Vining [179] classified landscape evaluation methods into five conceptual models: ecological, formal aesthetic, psychophysical, psychological, and phenomenological. Slightly earlier, Zube at el. [180] had suggested a similar classification scheme, in which there are four paradigms of landscape perception: expert, psychophysical, cognitive, and experiential; it is worth noting that, according to Zube at el., the ecological and formal aesthetic models (of Daniel and Vining’s classification scheme) are coalesced in the expert paradigm, while the term ‘landscape perception’ is used interchangeably with the term ‘landscape evaluation’. Another classification scheme regarding the evaluation of scenic beauty of natural resources, which was suggested somewhat later, grouped the relevant methods into three major categories: descriptive inventories, public evaluations, and economic analyses—with both quantitative and non-quantitative sub-methods embedded within each category.

In the last four decades, there has been a plethora of research studies focusing on how to register and (quantitatively/qualitatively) model landscape evaluation (assessment), resorting to the opinions and ratings of participants/correspondents who observe landscape scenes (e.g., photographs, simulations, videos, and in situ visits), by employing several empirical techniques for extracting their preference regarding the viewed landscape scenes, such as interviews or questionnaires, and developing (mathematical) models of visual (semi-)quantitative analysis (e.g., [54,55,56,57,58,59,60,61,62,63,64,65,66,67,68,69,70,71,72,73,74,75,76,77,181]).

An early example of such empirical research was conducted by Kellomäki and Savolainen [68]. They assessed (i.e., evaluated) the scenic value of selected forest stands using questionnaires (concerning both the actual stands and photographs of the same stands), in which participants expressed their scenic (landscape) preference by assigning an adjective—from a given set of adjectives (e.g., inspiring, ugly, repulsive, attractive, etc.)—indicating their positive or negative attitude. Through this research, they showed that mature stands of moderate density (e.g., coniferous stands with intermixed birches) had the greatest scenic value. In the same vein, Arriaza et al. [60] assessed the visual quality of agricultural landscapes in two Mediterranean rural areas (Southern Spain) by means of direct and indirect landscape evaluation techniques; the landscape scenes used in this research study were photographs including man-made elements, agricultural fields, natural parks, etc. Through the direct technique, the agricultural landscapes were ranked by carrying out a public preferences’ survey; through the indirect technique, the contribution of the elements/attributes contained in the photograph to its overall scenic beauty were weighted via regression analysis. In total, they showed that the perceived visual quality increases, in decreasing order of importance, with the degree of wilderness, the presence of well-preserved man-made elements, the percentage of plant cover, the amount of water, etc.

In a more generic aspect, in the relevant research studies for evaluating landscape public preferences based on specific landscape elements/attributes, it has been empirically evidenced that the presence of water (e.g., [55,58,60,77,182], vegetation (woody vegetation (i.e., forests), in particular) (e.g., [55,59,64]), topographically prominent features and mountains (e.g., [54,58]), and elements of (wild) nature (e.g., [60,65]) are positively evaluated. In quantitative terms, it is the simple presence of these elements that improves landscape preference and not their dominant presence ([59,73,74]); even more explicitly, the increase of the representation rate of some elements (e.g., vegetation) within a landscape scene can decrease the perceived preference [73], since it is often the spatial heterogeneity (diversity) of the different landscape elements that adds complexity to the landscape and further ensures the increased landscape attractiveness (high ratings and preferences) ([57,73,183]). Other relevant research papers examine and quantify the effect of the distance of landscape features (elements) on people’s visual satisfaction—when these elements are viewed through (artificial) windows [22]; the results show that both the visual content and the viewing distance matter: people are generally more satisfied when landscape elements are far away from the window viewpoint, while this fact particularly applies for urban elements and not for nature [22].

The recognition of aesthetics and the related impacts on the scenery has gradually led to the inclusion of visual impact assessment (VIA) as part of the broader and well-established practice of environmental impact assessment (EIA) [184] (p. 1006). Thus, visual impact assessment (VIA) for landscapes, where specific rather intrusive human activities (e.g., mining, renewable energy, industrial installations, etc.) potentially decrease public preferences for landscapes, has been embedded in the recent landscape evaluation literature (e.g., [74,185,186,187,188,189,190,191,192]). In the context of VIA, several research studies have been carried out. Dupont et al. [188] used photographic simulations of public facility constructions in rural landscape scenes and compared the outputs of saliency models, which compute the visual conspicuity on new constructions, to human assessments/preferences of the visual integration of these constructions obtained using photo-questionnaires. In the research of Misthos et al. [186], the Fuzzy Cognitive Mapping (FCM) method has been applied to explore the factors that affect the perceived nuisance caused by the impact of mining projects on the landscape; the collective conceptual FCM model being developed by a team of experts in mining and landscape engineering aided in identifying and quantifying the factors interacting in the “mining-landscape-society” system. Recently, Huai and Van de Voorde [72] harnessed a plethora of online reviews for several urban parks in Shanghai and Brussels, and applied Natural Language Processing (NLP) methods to classify the reviews into positive and negative ones and to identify which environmental (landscape) features contribute to positive and negative park perceptions (i.e., evaluations) in these two cities. Research studies such as [193,194] have implemented machine learning (ML) techniques on human ratings of public space (urban landscapes) form street-level imagery with an aim to predict human perceptual indicators (e.g., lively, wealthy depressing, etc.) for new urban landscapes [193], or to identify objects (landscape elements) that explain differences in ‘perception’ of safety among observers [194].

As previously stated, visual landscape assessment/evaluation from the egocentric perspective is a process that is distinct from landscape perception but not totally independent from it. For a landscape (scene) to have the potential to be evaluated (e.g., to be rated as attractive or disturbing), a set of landscape elements should have at least been perceived [23]. The vast majority of the abovementioned research papers assume that an array of specific elements is visually perceived within landscape scenes and, on the basis of their observations, they rate/evaluate the landscapes as a whole. In experimental studies of eye movement recording and analysis, it can be further proved whether and quantified to what extent the visual attention of observers focuses on these elements. Thus, from the viewpoint of VIA, “visual perception and attention constitute the necessary conditions for further addressing and assessing the visual impact or nuisance in mining landscapes” [29] (p. 622). In other research studies which employ other experimental techniques, namely fMRI and EEG, it is not always clear whether the registered data (e.g., metrics) while observing landscape scenes refer to modalities of visual perception (sensation) or evaluation (implicating cognitive/emotional aspects, as well).

In this sub-section, approaches, methods, and techniques regarding the visual landscape were presented, putting a dividing line between those dedicated to visual perception and those referring to evaluation. Yet, in any of these cases/modalities, landscape (regarding either its perception or its evaluation) was conceptualized from the egocentric perspective.

However, the egocentric perspective—as exclusively primary and dominating as it may seem—is not the only perspective, especially when it comes to landscape research. The landscape ‘inside the brain’ of a human and within the visual field of an observer/perceiver (egocentric perspective) is detached from—but somehow linked to—the landscape ‘out there’ (exocentric perspective). Due to this distinction, two divergent approaches and also two divergent traditions have been established as well. In the following sub-section, these two approaches are concisely adduced while aspects regarding the landscape conceptualized from the exocentric perspective are more extensively analyzed.

## 3. Descriptive and Normative Approaches in the Framework of the Two ‘Divergent’ Perspectives for Conceiving/Managing Landscape

### 3.1. The Two ‘Divergent’ Perspectives for Landscape: Egocentric vs. Exocentric

Fifty years ago, Rimbert [195] (pp. 234–235) suggested a generic classification for conceiving and conceptualizing landscapes. According to this classification, there are two essential landscape approaches or perspectives:

- The one that “apprehends the individual or the human as the starting point”; this approach refers to the philosophical attitude that places the *self* or the humans at the center of the world; according to this approach “what each individual directly perceives is not a neutral space, but rather an imaginary sphere of personal signs and signals”;
- The other that “considers space as an object of observation”; this perspective pertains to the philosophical reflection of the *Cartesian extension* whereby the adopted attitude is that of “an observer that is voluntarily detached from the space-object”.

In this ‘dipole’, there exist two poles/perspectives: the subjective egocentric and the objective exocentric. At the first pole, the subjective experience of each individual is shaped via the egocentric (i.e., human) perspective. Methods, techniques, and metrics for registering and modeling the subjective experience; i.e., human perception and evaluation of the visual landscape have been presented in the previous sub-section. In this sub-section, methods, techniques, and metrics for modeling landscapes regarding the second pole (exocentric perspective) are described.

As previously mentioned, from an egocentric (or ‘anthropocentric’) perspective, landscape is ‘‘an area, as perceived by people, whose character is the result of the action and interaction of natural and/or human factors’’ (European Landscape Convention (ELC) definition) [124] or “a geographical area, characterised by its content of observable, natural and human-induced, landscape elements” [196,197] (p. 559). Landscape elements are “natural or human-induced objects, categories or characteristics, including ecosystem types, which are observable at landscape scale” [197].

According to Jones et al. [198] (p. 210), the ELC landscape conceptualization contrasts with how landscape is conceptualized and typically defined by Landscape Ecology as “a tangible area of a certain given size and/or related to a certain spatial scale, with specific, pre-defined characteristics”. While the ELC definition emphasizes the culturally-driven manner based on which each human observer ‘constructs’ landscapes from the material environment [198,199], in “Landscape Ecology a landscape is [rather] defined from the viewpoint from different species including humans” [198] (p. 210) or from the ‘view from above’.

Therefore, instead of ceding the primacy to the subjective, egocentric human perspective, one can adopt another approach which lies in studying the landscape itself, as the latter is represented in an exocentric (i.e., vertical) perspective, by quantitatively analyzing its elements and its ‘intrinsic’ properties [21]. As previously mentioned, from the viewpoint of Landscape Ecology landscapes are regarded as extensive, heterogeneous land mosaics composed of clusters of interacting ecosystems which recur in similar form throughout [82,86,198]. Landscape Ecology explicitly studies how ecological processes—the behavior and distribution, i.e., function, of organisms—are affected by landscape pattern [7,200,201]. What is explicitly and quantitatively studied refers to both *composition* (“how much there is of a particular component”) and spatial *form* or *configuration* (“how it is arranged”) [83] (p. 4).

The explicit manner whereby landscape pattern is studied is based on geospatial quantitative analyses employing metrics and/or indices (The terms ‘landscape metrics’ and ‘landscape indices’ are often used interchangeably, as there are no specific rules for when to use the one term over the other [202]. The term ‘landscape indices’ appears more frequently when used in a broader sense, while the term ‘landscape metrics’ appears more frequently for metrics calculated within computer programs and software packages (e.g., FRAGSTATS), and it has also become the prevalent term in the last two decades [202]. For simplicity, the term ‘landscape metrics’ will be used exclusively in the rest of the paper.) that assign values to the planimetric (and vertical) heterogeneity of landscape’s constituent elements and/or patches [7,8,83,87,88]. Expressing spatial heterogeneity by means of metrics is needed in order to establish quantitative relationships between spatial patterns and ecological processes and spatial patterns [202]. In general, the metrics either for quantifying landscape composition or configuration/structure occur by initially either taking into consideration individual grid *cells* or by identifying and extracting land *patches* in classified/categorized georeferenced images or maps. A *patch* is theoretically defined as a relatively uniform surface area that differs from its surroundings in nature or appearance [83,86]. In practice, patches can be areas of “similar vegetation or land cover” [83] (p. 72) or homogeneous habitat types, while a more technical definition for algorithmically identifying patches on a landscape represented by a rasterized or gridded format consists in delineating “a contiguous group of cells of the same mapped category” [83] (p. 106).

As Uuemaa et al. [202] state, a wide variety (hundreds) of metrics have been developed for quantifying categorical map patterns in terms of landscape composition and spatial configuration. Landscape composition metrics—such as relative richness, areal percentage (%) of specific (land, vegetation, habitat) cover types, or Shannon’s diversity index, etc.—and landscape structure metrics—such as contagion (of cell cover types), patch size, shape index of patches, or proximity among patches of the same cover type, etc.—have been employed in a plethora of case studies. These metrics appear in a variety of different themes and applications in the field of landscape ecology such as biodiversity and habitat analysis (e.g., [203,204]), urban landscape pattern (e.g., [205,206]), aesthetics of landscape, (e.g., [207,208]), etc.—according to the way [202] summarize and classify the pertinent research studies.

Aside from the multitude of different domains where landscape metrics are used, there are several software packages and tools dedicated to the computation of these metrics. Thus, the most common usage of landscape metrics refers to indices developed for quantifying categorical map patterns [202] by using these software packages and tools. In the last three decades, stand-alone software packages specifically designed to compute a variety of landscape metrics for categorical map patterns have been developed, such as FRAGSTATS released in different versions [88,209,210]; add-on modules or plug-ins into existing GIS and image processing/analysis software, including module patterns in IDRISI and Patch Analyst in ArcGIS, LecoS in QGIS [211], Arc_LIND in ArcGIS [212], etc.; or open source programming libraries such as PyLandStats for automatedly computing landscape metrics in interactive environments [213].

### 3.2. Descriptive Approach: Classification/Characterization Process in Landscape Character Assessment

Landscape Ecology has significantly aided—with the operationalization of its definitions—in creating and defining “one or several ‘landscape categories’ that are to be subjected to management or other actions” [197]. In essence, Landscape Ecology enables the classification/characterization and mapping of landscape at a local, national, or continental (e.g., E.U.) level, making use of the exocentric perspective.

The fundamental concept in terms of landscape management and policy making is *Landscape Character Assessment* (LCA). The basic principles and terminology of LCA (with reference to England and Scotland) have been introduced by Swanwick [90]: “LCA is concerned primarily with landscape character, rather than with landscape quality or value”, while character itself refers to “a distinct, recognisable and consistent pattern of elements in the landscape that makes one landscape different from another, rather than better or worse” [90] (p. 8). In this sense, the process of characterization includes the identification of *landscape character types* (generic in nature) and of *landscape character areas* (unique/discrete areas of a particular type), while the end product of characterization is typically a *map* that classifies a geographic region in landscape types/areas [90].

As a consequence, according to this process, landscapes can be objectively described and analyzed at the sub-national, national, or super-national level, without the implication of human presence/perception and evaluative judgements. To this end, objective/quantitative criteria, methods, and techniques have been developed in order to assess the character of landscapes mainly in Europe or elsewhere [89,90,91,92,93,94,95,96,197]. Such projects have been implemented primarily by employing a GIS-based procedure. Fundamental aspects in this procedure include the choice of key parameters (e.g., topography, parent material, land use, etc.) that are readily available as geospatial data layers and the systematic and traceable combination of these layers into one overarching landscape concept [95]. For instance, European Landscape Classification (LANMAP), as described by [89] (p. 100) “has been used in the initial phase of a Landscape Character Assessment, in order to facilitate the analyses of the structure and pattern of landscapes”, and further aids in covering “the need for a common and geo-referenced classification system of landscapes for Europe”. The final result is the European Landscape Map, LANMAP, a Pan-European geo-referenced, multi-resolution (multiple levels) landscape classification scheme based on geospatial data on climate, altitude, parent material, and land use, mainly derived from satellite imagery [89]. Other similar attempts for classifying the landscape character have been conducted for other regions, e.g., New Zealand [93,94,96]. For an extensive review of other worldwide LCA approaches see [196,214].

As previously mentioned, LCA “is primarily concerned with documenting landscape character rather than assigning quality or value” [91]. The act of documenting is a value-free process and a rather objective and quantitative one. However, there are two stages in LCA: the characterization, “which is relatively value-free and is concerned with identifying, classifying and describing areas of distinctive character”, and the making-judgements (evaluation) stage “to inform particular decisions, which may use one or a combination of approaches depending on the purpose of the exercise” [90] (p. 16).

### 3.3. Normative Approach: Evaluation Process in Landscape Character Assessment

The process of characterization or classification provides the central, rather objective framework on which subsequent evaluative judgements about landscape character are to be based [90]. Therefore, assigning value or quality to the landscape (i.e., evaluation) is a separate, normative process which requires the involvement of human cognition and judgement. This process engages, in principle, subjectivity.

In the literature, and in practice, it has been shown that attempts for landscape evaluation adopting the exocentric perspective in a direct manner is a tricky venture. For instance, Tveit et al. [215] developed concepts (dimensions, attributes, and indicators) for analyzing the *visual* character of landscape towards further evaluating the landscape change. This indirect manner of proceeding to landscape evaluation from the exocentric perspective is shown in that “each of these [selected] concepts focuses on different aspects of the landscape important for *visual quality*, where *visual quality* is an holistic experience of them all” [215] (p. 229). As they explicitly put it, “the visual concepts presented are used to *describe* different characteristics of visual landscapes, rather than presenting a *normative value* for *visual quality*” [215] (p. 229). In the same vein, Ode et al. [216] claim that a visual assessment that is objective in its nature can form a robust basis for the subsequent evaluation of landscape visual quality; they suggest an approach for describing visual concepts through measurable visual indicators and for further linking visual indicators to theories of landscape perception and preferences (aesthetics). On the contrary, in a very recent review article, Lothian [217] concludes that the vast majority of academic research and institutional regulations, particularly in Britain and Europe, fixate on the landscape character and not on landscape quality; due to the allegedly intricate manner of measuring landscape/scenic quality (which also encompass subjectivity), landscape character has become the objectively-measured substitute instead. Yet, this pronounced focus on landscape character alone, and the simultaneous neglect of quality or scenic value, “loses the plot” [217] (p. 451).

As it may appear, there is a rather inherent incompatibility of proceeding to landscape evaluation from the exocentric approach without resorting to the *visual* landscape—i.e., to the landscape as it can be *visually* perceived via the egocentric perspective. This apparent incompatibility “between the ELC landscape definition” (egocentric perspective) and ‘natural science-based landscape definitions’ (exocentric perspective) has been clearly noted by Erikstad et al. [197] (p. 11); besides, this lack of compatibility poses a challenge for both scientific research and planning processes [197]. The ‘biophysical landscape concept’ introduced by Simensen et al. [196] for methods concerned with the material content (i.e., natural and man-made elements) of the landscape is aligned to ‘natural science-based landscape definitions’ and is compatible with “basic typification, characterisation and mapping of landscapes” which is linked to important practical aspects for landscape management [197] (p. 11). Yet, the stage of evaluation transcends the landscape characterization stage which solely “concentrates on what makes one area different from another” [214] (p. 53) by resorting to the general (intrinsic) properties of landscape *per se*. Landscape evaluation entails the expression of judgements about landscape character, further “leading to decisions concerning the management, planning, and protection of the various landscape types/areas” [214] (p. 53). Without the verbalized human judgements about the landscape, the characterization of landscape can remain just another useful but inactivate piece of scientific evidence; instead, the scientifically informed evaluative judgements of active citizens can promote and enable the practical implementation of planned actions towards the proper management and protection of landscape types or areas. Thus, landscape evaluation must encompass human perception and citizen participation in processes leading to landscape management [197]. Meanwhile, the ‘holistic concept’ of landscape has to be adopted, including “human perception and cultural relations to areas” [196].

In order to avoid this impasse (see [214,218] for the ‘debates’ regarding objective vs. subjective and quantitative vs. qualitative)—since clear-cut natural science-based landscape definitions limit the inclusion of human perception and citizen involvement [197]—a viable solution lies in utilizing LCA for objectively/quantitatively specifying and delineating the different geographical areas to be further subjected to the scrutiny of subjective human perception and evaluative judgements. In other words, when it comes to landscape evaluation for pragmatic decision making pertaining to land management and planning, one has to initially comply with the current “classification of landscape description units-LDUs” [214] (p. 53), and then resort to judgements of the public which rate/rank the different LDUs based on perceptual, cognitive/emotional, or functional criteria. Yet, rating/ranking the landscape can be attained by means of encompassing the subjective experience of these LDUs in some way.

In their recent conceptual article, Terkenli et al. [214] summarize and classify a wide variety of practiced or proposed LCA methodologies according to the way they negotiate the interplay between objective and subjective landscape dimensions, by employing quantitative or qualitative approaches and data. One of the ‘most subjective’ and ‘qualitative’ approaches is the one adopted by Scott [219] using LANDMAP for identifying distinctive landscape areas on the one hand, and properly selected photographic material conveyed via household questionnaires/focus groups for evaluating public perception; the results provided important insights into public perception, allowing for particular landscape types and areas to be evaluated—quantitatively and qualitatively. Another ‘very subjective’, but also ‘very quantitative’ approach has been adopted by Atik and Karadeniz [220] which integrate the ELC definition into the LCA methodology in order to evaluate the importance and to further identify landscape functions and potentials for different land use of two landscape areas in Turkey based on the evaluation of different biophysical layers and without neglecting the subjectivity of visual characteristics.

In other similar approaches [221], various types of landscape characters in specific regions of Turkey are examined in order to determine the visual quality and visual preferences of these different landscape types; nine different landscape character types conveyed via representative photographs/images were evaluated (in terms of landscape scenic beauty, and other parameters, such as vitality, safety, impressiveness, degradation, worth being protected, etc.) by a large number of participants using a questionnaire-based survey. In the recent paper of Criado et al. [222] (p. 6395), landscape evaluation is treated as a complementary tool in the assessment of the environment; after identifying the characteristic/distinctive landscape units in urban areas of Spain, the landscape is ‘diagnosed’ based on an objective and quantitative GIS-based evaluation of the landscape situation in each of the identified landscape units, “according the extension (ha) and percentage (%) of each degree (very high, high, moderate, low and very low) of quality, fragility or need for protection in each unit”.

## 4. Discussion

### 4.1. Summarizing the Dualities of Landscape: A Critical Examination

The essential framework for presenting this review article is constructed based on two dualities: the two modalities of human perception and evaluation and the two ‘divergent’ or alternative perspectives—egocentric and exocentric—under which landscape can be conceived. Through scrutinizing a very extensive body of literature, we primarily focus on different manners to model the fundamental activities related to landscape under the two human modalities and the two geometric perspectives. Table 1 acts as a ‘guide-map’ which concisely portrays the four main activities related to landscape experience and/or management: (a) perception and evaluation from the egocentric perspective; (b) characterization and evaluation from the exocentric perspective.

In general, Table 1 is comprised of two columns referring to the four different activities under the two perspectives and of six columns that refer to some useful attributes, namely meaning, indicative keywords, ‘stimulus’, ‘sensor’ type, metric type, and indicative literature. Each of these columns/attributes provides descriptions or information that are (potentially) valid or expected to be compatible with each activity; for each activity, a collection of pertinent literature is also included. This meta-analysis is not eliminative but rather an attempt to demarcate the different activities based on indicative (or type of) information suitable for each of the six selected attributes.

Recent review/conceptual articles provide comprehensive overviews of important issues, including methods for landscape characterization and mapping [196], evaluation methodologies, technologies and recommendations on landscape visual quality [78], and the interplay of objectivity and subjectivity in LCA [214]. However, the first article [196] systematically reviews methodologies that almost exclusively pertain to LCA. According to our meta-analysis, the methodologies reported in [196] fall under the activity of characterization and under the exocentric perspective. The second article [78] is an extensive review of methodologies and technologies that focus almost exclusively on landscape evaluation. Based on our meta-analysis, the methodologies and technologies mentioned in [78] refer primarily to the evaluation activity under the exocentric perspective; nevertheless, in [78] there can be found ‘stimuli’ that, according to Table 1, fall into the exocentric perspective. This is an issue that can cause confusion but also raises an important and interesting topic that is discussed more thoroughly and constructively in the following sub-section. The third article [214] delves into the intricate issue of how objective-subjective approaches and quantitative-qualitative constituent parts interweave in existing or proposed LCA methodologies. The general approach adopted in [214] is mostly aligned to the exocentric perspective’s characterization activity, with a tendency to integrate the exocentric with the egocentric and the descriptive with the normative approach. The latter raised challenges, i.e., integrating the descriptive with the normative landscape approaches and perception with evaluation of the visual landscape is also discussed in the following sub-section. Lastly, as one can observe, the last column, indicative literature, is rather meager for the case of landscape evaluation under the exocentric perspective. The ‘incompatibility’ of evaluating landscapes from the exocentric perspective—in the context of LCA—has been already revealed in the previous section. As Lothian [217] has ascertained in his review article regarding “visual resource stewardship”, estimating the subjective scenic quality and scenic value (i.e., landscape evaluation) has been reduced, by and large, to the objective description of landscape character.

### 4.2. Bridging the Gap between the Two Dualities

#### 4.2.1. How to Integrate the Exocentric with the Egocentric Perspectives

In the context of landscape experience and management, several approaches, methods, and techniques have been suggested or adopted for integrating the two geometric perspectives of conceiving landscape (e.g., [20,21,23,97,101,223,224,225,226,227,228]). In general, the most useful analytical computing procedure implemented in this context is the (GIS-based) visibility or viewshed analysis, with a focus on the human (egocentric or 3D) perspective (e.g., [20,98,101,224,227,229,230,231,232,233].

Dramstad et al. [97] tested whether map-derived indices of landscape composition/structure (exocentric perspective) are correlated with visual landscape preferences based on “on-the-ground photography” (egocentric perspective); the results show that certain landscapes have relevance in relation to landscape preferences in certain landscape types. The conceptual article of [226] attempts to establish a bridge between visual (egocentric) landscape indicators (derived from [215,216]) and ecological (exocentric) landscape indicators; from this integrative approach, a set of indicators capturing important visual and ecological landscape dimensions has been identified. Three other, more recent, significant approaches for transferring the content of the landscape from the exocentric perspective to the egocentric one have been suggested and implemented by Domingo-Santos et al. [232], Nutsford et al. [101], and Brabyn [224]: in the first two [101,232], tools and methods were developed for extending the standard viewshed (exocentric) to represent the visible landscape as perceived by humans (egocentric), via the concepts of visual exposure and visual significance, respectively; in the third one [224], another methodology was also developed whereby the experience of the landscape character while walking a track (egocentric) is generated (through “experions”) by combining GIS viewshed analyses with GIS-based landscape character classification (exocentric).

One of the most informative approaches has been developed by Schirpke et al. [20]: in their research study, an innovative GIS-based modeling approach which integrates objective methods with perception-based methods is implemented. In practice, by utilizing viewshed analyses and based on separate viewpoints, the visible landscape’s spatial patterns were analyzed by means of a variety of landscape metrics’ components (objective/exocentric), while these components were statistically related to subjective scenic beauty values (subjective/egocentric). Finally, in the research work of [23], a multi-parametric geospatial (GIS-based) model was developed for assessing the landscape impacts from surface mining. This model was implemented (in line with [227]) by geospatially transferring the measurement of these parameters through certain indices and quantitative criteria related to landscape characterization and scenic value—such as size of quarry, complexity/diversity of landscape, naturalness of the landscape (exocentric), into their perceived or apparent counterparts—i.e., apparent size of quarry, apparent/perceived complexity/diversity of the landscape, perceived naturalness (egocentric) by making use of viewshed and other GIS-based analysis.

Such approaches illuminate the way towards bridging the conceptual and methodological gap of conceiving and ‘manipulating’ the landscape experience and management. Aside from bridging the gap between the two geometric perspectives, some of the previously described approaches (e.g., [20,23,97]) are also directed towards integrating the descriptive with normative and/or the perception with the evaluation modalities, as well. In the following, there are some attempts specifically towards this direction.

#### 4.2.2. How to Integrate Descriptive with Normative and Perception with Evaluation (Modalities)

As previously presented, many research studies aim to model the visual perception or the evaluation of a set of landscapes or landscape types (e.g., rural, urban, forest, mountainous, and mining). To this end, different (types of) landscapes are utilized as visual stimuli. There is another set of research studies that seek to establish an association between the (descriptive) properties of a stimulus, i.e., the visual scenes of landscapes, and the way these scenes are (normatively) evaluated. For instance, ref. [234] explored the relationship between three objective landscape indicators of naturalness—which were manipulated in landscape photo-simulations—and subjective landscape preferences, while the results revealed a strong relationship between subjective preferences and with the two (of the three) landscape indicators of naturalness. Another recent article [235] investigated the association between the objective geometric properties and the complexity of nature landscape scenes and the subjective aesthetic preferences; by utilizing Alpine landscape photo-simulations in which manipulations were applied to alter their complexity, it was shown that landscape scenes with organized complexity were the most aesthetically appealing.

**Figure 6:**
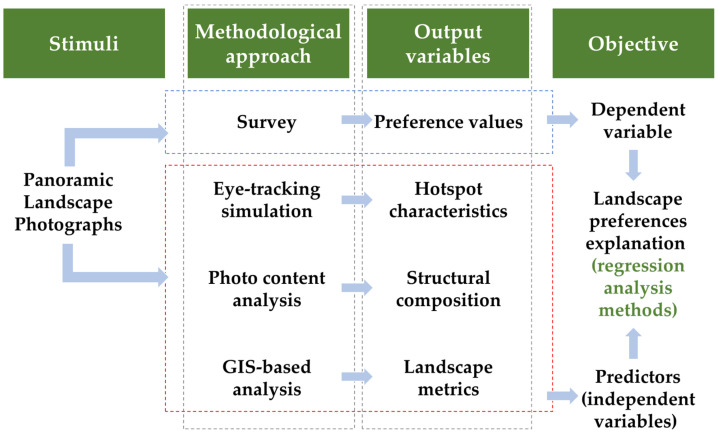
Overview of the developed methodology utilizing a combination of eye-tracking simulation, photographic content analysis, and geospatial analysis to extract the predictors (independent variables) in order to explain people’s preferences of alpine landscapes (dependent variable) being collected by means of surveys based on panoramic landscape photographs. The figure has been created according to [100].

A very promising article that intertwines landscape perception with landscape evaluation from both perspectives is authored by Schirpke et al. [100] which developed a pioneering methodology to compare the information type obtained through eye-tracking simulation with landscape preferences. More specifically, they compared information in the following ways: (1) Based on a large number of (egocentrically-captured) landscape photographs, representing major Alpine landscape types, using visual perception eye tracking hotspot-based indicators (i) and variables describing the content of the landscape photographs (ii); (2) Based on (exocentric-based) landscape metrics derived using the visible (viewshed-delineated) landscape’s spatial patterns (based on the research of [20]). Figure 6 schematically summarizes the methodology being developed and implemented in [100]. Although the analysis of eye-tracking hotspots is limited in generic explanations across different landscape types, it is a great step forward.

Such attempts clear the ground for other, more challenging, attempts that aim at predicting or anticipating the visual perception and/or evaluation of a random landscape, given a large set of landscape scenes. As previously mentioned, there are several articles that use machine learning (ML) techniques to evaluate landscapes (e.g., [193,194,236]). However, the acute problem of potentially anticipating the human ratings (i.e., evaluation) of different landscape types is not addressed. In a recent conceptual article [237], a methodological framework is suggested to bridge the gap between the following: (i) The inherent properties of different landscape types’ photographs, as these properties are described by various objective metrics/indices (e.g., image complexity/entropy (e.g., [13,238], or landscape indices); (ii) The properties of human perception while observing these landscape photographs, as these properties are objectively expressed through various eye movement metrics and more composite indices (e.g., [149,239]). The expected outcomes of such a methodological approach are the potential prediction of the dominant viewing patterns and the subsequent evaluative preference ratings of each landscape (type) scene, by applying ML (e.g., Artificial Neural Network) techniques on these two types of metrics/indices ((i) and (ii)).

### 4.3. Concluding Remarks and Future Outlook

Through an extensive literature review, existing approaches on visual landscape quantification and modeling have been identified in a two-fold manner, based on the following: (1) Whether the focus is on landscape perception or landscape evaluation; (2) Whether the landscape is conceived from the egocentric or from the exocentric perspective. The main aim of this review was to provide a demarcation approach within the vastness of the relevant literature, in theoretical, methodological, practical, and technical terms, for the four distinct but interrelated activities pertaining to visual landscape experience/management (perception, characterization, and evaluation). Moreover, frameworks that tend to integrate these distinct but interrelated activities were also promoted.

As shown, research regarding landscape experience and management quickly expands but so does the confusion. However, some recent approaches are directed toward the integration of different perspectives and modalities of landscape experience by employing methods, techniques, sensors, and metrics from different disciplines and traditions. In our opinion, it appears necessary to resort to integrative, interdisciplinary approaches if we are to better understand how the landscape is imprinted on visual perception and potentially leads to more elaborate and novel outcomes such as anticipating human viewing behaviors or even landscape preferences. Given that landscapes have been the field of survival for our ancestors in the past, but also a valuable resource that can ensure the well-being of individuals and of entire societies in present times, it is of great importance to establish theoretical, methodological and, technical tools towards modeling the actual visual landscape ‘out there’ and ‘inside the minds’, and modeling ‘what people see’ and ‘what people appreciate’, while finding valid interconnections among them. We believe that a rigorous, evidence-based, and righteous approach towards landscape management, protection, and decision-making should be based on integrative and interdisciplinary frameworks that fuse the measurement/quantification of the landscape’s physical properties with the actual perception and preferences of a wide portion of the society, making the most of a wide spectrum of well-suited and advanced sensor-based technologies (e.g., eye tracking, fMRI, and remote sensing imagery).

## Figures and Tables

**Table 1:** Meta-analysis (classification) of some fundamental attributes based on the four activities related to landscape experience/management under the egocentric and exocentric perspectives.

Perspective	Landscape-Based Activity	Meaning	Indicative Keywords	‘Stimulus’ Type	‘Sensor’ Type	Metric Type	Indicative Literature
egocentric	perception	how humans visually perceive a landscape (scene) during everyday observation	visual perception, visual attention, etc.	photos, simulations, and actual landscape	eye trackers (ET), fMRI, and EEG	objective ET-, fMRI-, and EEG-based indices	[24,25,26,27,28,29,30,31,32,33,34,35,36,37,38,39,40,41,42,43,44,45,46,47,48,49,50,51,52,53]
	evaluation	how humans rate a landscape (scene) or express their preferences about a landscape (scene) during everyday observation	visual preferences, visual quality, landscape aesthetics, etc.	photographs, stimulations, and actual landscape	questionnaire-based (for a wide array of utilized techniques see [78])	subjective ratings or rankings(+ objective ET-, fMRI-, and EEG-based synthetic indicators)	[54,55,56,57,58,59,60,61,62,63,64,65,66,67,68,69,70,71,72,73,74,75,76,77]
exocentric	characterization	how a certain geographic region is classified into different, distinct landscape areas based on its intrinsic properties	landscape change, LCA areas, LDUs, etc.	geospatial data: remote sensing (e.g., satellite) imagery, classified (LULC *^1^) maps, etc.	GIS-based	objective indicators based on landscape ecology indices, LCA typologies, etc.	[89,90,91,92,93,94,95,96,203,204,205,206,207,208]
	evaluation	how different, distinct landscape areas of a certain geographic region are rated based on their intrinsic (or extrinsic) *^2^ properties	landscape quality, landscape aesthetics, etc.	geospatial data: remote sensing (e.g., satellite) imagery, classified (LULC) maps, etc. (+photographs, stimulations, and actual landscape)	GIS-based (+questionnaire-based)	objective/subjective ratings or rankings based on the intrinsic (or extrinsic) quality of each landscape area	[219,220,221,222]

*^1^ Land Use/Land Cover. *^2^ Typically, the ratings are based on quantitatively expressing the (intrinsic) properties of the landscape itself, usually in the form of layers (e.g., terrain slopes and vegetation cover), and then ranking them according to some ‘objective’ criteria (e.g., mild/moderate/intense terrain slopes, etc.), to finally determine the ‘quality’ of each landscape area. In a few other cases, (extrinsic) evaluative judgments about the ‘quality’ of each landscape area are made by human respondents.

## Data Availability

Not applicable.
